# In infertile women with subclinical hypothyroidism, with or without thyroid peroxidase antibodies, serum TSH during pregnancy follows preconception values and thyroid hormones remain stable

**DOI:** 10.1093/hropen/hoad038

**Published:** 2023-10-09

**Authors:** C De Geyter, L Matt, I De Geyter, R Moffat, C Meier

**Affiliations:** Reproductive Medicine and Gynecological Endocrinology (RME), University Hospital, University of Basel, Basel, Switzerland; Reproductive Medicine and Gynecological Endocrinology (RME), University Hospital, University of Basel, Basel, Switzerland; Reproductive Medicine and Gynecological Endocrinology (RME), University Hospital, University of Basel, Basel, Switzerland; Reproductive Medicine and Gynecological Endocrinology (RME), University Hospital, University of Basel, Basel, Switzerland; Division of Endocrinology, Diabetes & Metabolism, University Hospital Basel, University of Basel, Basel, Switzerland

**Keywords:** thyroxine, thyrotropin, thyroid peroxidase antibodies, subclinical hypothyroidism, infertility diagnostics, pregnancy, fertility diagnostics, preconception

## Abstract

**STUDY QUESTION:**

How does subclinical hypothyroidism, defined in infertile women during preconception by thyroid-stimulating hormone (TSH) >2.5 or >4.5 mIU/l, with or without thyroid peroxidase antibodies (anti-TPO) >100 IU/ml, impact thyroid hormone levels during pregnancy and after birth?

**SUMMARY ANSWER:**

During pregnancy, TSH levels remain similar to those in preconception, even with supplementary thyroxine, whereas the serum levels of anti-TPO progressively decline.

**WHAT IS KNOWN ALREADY:**

Overt hypothyroidism impacts both pregnancy and offspring but randomized clinical trials and cohort studies failed to detect the benefit of treatment with thyroxine in cases with low-threshold TSH or with anti-TPO during pregnancy.

**STUDY DESIGN, SIZE, DURATION:**

First, the prevalence and reproducibility of two candidate cut-off levels of subclinical hypothyroidism in a cohort of 177 infertile women was compared with 171 women not aiming for pregnancy. Second, the impact of distinct setpoints of TSH in preconception (with or without anti-TPO) was monitored during pregnancy in 87 previously infertile women by high-frequency monitoring of thyroid function. Both studies were carried out from 2007 to 2019.

**PARTICIPANTS/MATERIALS, SETTING, METHODS:**

Reproducibility and prevalence of subclinical hypothyroidism were examined in infertile women presenting in the fertility care unit of an academic institution. Women not aiming for pregnancy participated as controls. In both groups, TSH and anti-TPO were measured two times on different occasions. In addition, a group of previously infertile women with known preconception setpoints of TSH (with or without anti-TPO) were followed up prospectively throughout pregnancy and after birth. During pregnancy, serum was sampled weekly until Week 12, then monthly until delivery, and once after birth. Only cases with preconception TSH >4.5 mIU/l were supplemented with thyroxine. After collection of all samples, the serum levels of anti-TPO and the major thyroid hormones were measured. Prolactin with known fluctuations during pregnancy was used as reference.

**MAIN RESULTS AND THE ROLE OF CHANCE:**

Measures of both TSH and anti-TPO at two different time points were accurate and reproducible. The odds of subclinical hypothyroidism in infertile women and controls were similar. During pregnancy, TSH closely followed preconception TSH levels, whereas serum levels of the thyroid hormones predominantly remained within or above (not below) the reference. Treatment of infertile women with preconception TSH >4.5 mIU/l with thyroxine resulted in higher free thyroxine (fT4) serum levels. The serum levels of anti-TPO declined as pregnancies evolved.

**LIMITATIONS, REASONS FOR CAUTION:**

The numbers of participants both in the prevalence study and in pregnancy did not reach the *a priori* estimated numbers. For ethical reasons, the patients with preconception TSH >4.5 mIU/l were treated with thyroxine. The findings apply to infertile women only.

**WIDER IMPLICATIONS OF THE FINDINGS:**

We propose to use >4.5 mIU/l as the serum TSH threshold for supplementing women with thyroxine before pregnancy. During pregnancy, fT4 may be the better marker to monitor thyroid function. The consistent decrease of anti-TPO antibody levels during ongoing pregnancies must be considered a protective element.

**STUDY FUNDING/COMPETING INTERESTS:**

The prevalence part of this study was supported by Merck-Serono, Geneva (TH006/EMR200007-603). The hormone measurements of the serum samples collected during the follow-up pregnancies were made possible by financial support of Roche Diagnostica (November 1721, 2017, Rotkreuz, Switzerland). I.D.G. was supported by a grant of the Repronatal Foundation, Basel, Switzerland. All authors declare no conflict of interest.

**TRIAL REGISTRATION NUMBER:**

Research Database of UniBasel, project no. 576691 (2007).

WHAT DOES THIS MEAN FOR PATIENTS? Reduced hormone output of the thyroid gland is a common finding in infertile women. This usually goes without any symptoms. During pregnancy, low thyroid hormone levels may cause miscarriage and other pregnancy-related complications. Reduced exposure to maternal thyroid hormones may also cause damage to the offspring. The concentration of the thyroid-stimulating hormone (TSH) is generally being used to screen for reduced hormone output of the thyroid gland. Over time, professional organizations have lowered the threshold of TSH for supplementation treatment with the main thyroid hormone, thyroxine. As a result, thyroxine has now become one of the most prescribed drugs during pregnancy and overtreatment has become commonplace. Recent clinical trials have raised doubts about the utility of low-threshold treatment with thyroxine during pregnancy.

We decided to evaluate systematically the accuracy and efficacy of screening the thyroid gland prior to, during and after pregnancy in infertile women.

Our findings showed that the standard thyroid screening protocol at two different time points before pregnancy was accurate and reproducible. The likelihood of thyroid abnormalities in infertile women and in women currently not aiming for pregnancy was similar. In a group of previously infertile women, the hormone output of the thyroid gland during pregnancy was found to be stable, even in cases with slightly reduced hormone output from the thyroid gland. We also demonstrate that the serum levels of autoantibodies against the thyroid gland systematically decline as pregnancies evolve.

Based on these findings, we propose to raise the threshold level of TSH for substitutional treatment of infertile women with thyroxine to avoid overtreatment.

## Introduction

Subclinical hypothyroidism is defined by elevated serum TSH levels in the presence of peripheral euthyroidism. It is the most common disorder of the thyroid gland, affecting 5–10% of women of reproductive age (Tunbridge *et al.*, 1977; Staub *et al.*, 1992; Surks *et al.*, 2004; Teng *et al.*, 2013; Hennessey and Espaillat, 2015). A plethora of both retro- and prospective studies has demonstrated that hypothyroidism is associated with complications during pregnancy and with neuro-developmental disorders of the offspring (Haddow *et al.*, 1999; Van den Boogaard *et al.*, 2011; Zhu *et al.*, 2021). Thyroid peroxidase antibodies (anti-TPO) may further aggravate the impact of hypothyroidism on the incidence of complications during pregnancy, including miscarriage and premature delivery (Poppe and Glinoer, 2003; Negro *et al.*, 2006). Screening of thyroid function is recommended as part of a fertility workup (Practice Committee of the American Society of Reproductive Medicine, 2015; Alexander *et al.*, 2017; Poppe et al., 2021), most particularly prior to treatment with assisted reproduction. Since the introduction of third-generation assays for the measurement of serum thyroid-stimulating hormone (TSH) levels in the 1990s (Spencer *et al.*, 1990), TSH has been used as a screening marker for the detection of hypothyroidism.

Conventionally, subclinical hypothyroidism was defined by mildly elevated TSH serum levels varying between 4.5 and 10 mIU/l (Surks *et al.*, 2004). This range was selected because of the higher risk of further worsening of thyroid function over time toward overt hypothyroidism (Wong *et al.*, 1981). Later, considerably lower threshold levels for subclinical hypothyroidism were advocated, such as 2.0 or 2.5 mIU/l (Dayan *et al.*, 2002; Wartofsky and Dickey, 2005). Although these lower threshold levels were later revoked, they are still recommended in infertile women prior to treatment with ART or in the presence of anti-TPO (Alexander *et al.*, 2017; Poppe *et al.*, 2021). Depending on the definition, 16–20% of women seeking fertility care may be classified as hypothyroid (Dhillon-Smith *et al.*, 2020) resulting in thyroxine becoming one the most commonly prescribed drugs for long-term use (e.g. 3.8%, Janett-Pellegri *et al.*, 2021). Up to 10–16% of pregnant women now receive thyroxine (Maraka *et al.*, 2017; Yu *et al.*, 2021). Overtreatment may, in up to 21% of treated individuals, lead to subclinical or overt hyperthyroidism (Parle *et al.*, 1993; Surks *et al.*, 2004; Janett-Pellegri *et al.*, 2021).

Various factors render treatment of hypothyroidism with thyroxine supplementation during pregnancy challenging. On the one hand, during pregnancy, both the enhanced renal excretion of iodine and the increased hepatic production of binding globulins exert more strain on the thyroid gland, while on the other hand, the rapidly rising serum levels of hCG support thyroid function by binding TSH receptors in the thyroid gland. In women diagnosed with overt hypothyroidism, the requirement for the administration of incremental substitutional thyroxine dosages during ongoing pregnancies has clearly been demonstrated (Alexander *et al.*, 2004). In contrast, in euthyroid women, the initiation of substitutional treatment based on mildly elevated TSH levels diagnosed during ongoing pregnancy was shown to be without beneficial effect (Casey *et al.*, 2017). The safety of low-threshold TSH substitution with thyroxine during pregnancy has also been questioned (Maraka *et al.*, 2017).

The dynamic endocrine changes during early gestation are crucial in the assessment of the adequacy of thyroid function. Only a few studies have analyzed longitudinally the changes in thyroid function during the various stages of pregnancy in women diagnosed with subclinical hypothyroidism: during early pregnancy (De Geyter *et al.*, 2009) and during ongoing pregnancy (McClain *et al.*, 2008; Yu *et al.*, 2013). No study has so far been carried out to trace the endocrine function of the thyroid gland longitudinally from preconception to pregnancy until after birth. The present study was designed to assess the effects of different preconception setpoint levels of TSH, in women with and without anti-TPO antibodies, on the endocrine performance of the thyroid gland during pregnancy and after birth.

## Materials and methods

The study protocol consisted of two parts. The first was a prevalence study to determine thyroid gland abnormalities in infertile women as compared to control women not aiming for pregnancy at the time of recruitment and to study the reproducibility of the thyroid function assessment based on TSH and anti-TPO. The second part was a prospective follow-up study of previously infertile women with a known preconception TSH setpoint through sequential measurements of the main endocrine parameters of the thyroid gland, including TSH and anti-TPO, during a subsequent pregnancy. The study was presented to and approved by the local ethics committee (EKNZ 39-2009/Req-2017-00960) and registered in the Research Database of the University of Basel: project no. 576691.

### Prevalence study

A total of 177 consecutive women presenting with infertility and willing to undergo two blood samplings within 2–4 weeks, together with an ultrasound examination of their thyroid gland, were recruited in the institute of Reproductive Medicine and Gynecological Endocrinology (RME) of the Women’s Hospital, University Hospital, University of Basel, Switzerland. The serum samples were taken before the initiation of any treatment of infertility. The control group consisted of 171 women, who were recruited through advertisements and posters in the close neighborhood of the university and of the University Hospital. Control women were selected because they were not aiming for pregnancy. To examine the diagnostic accuracy and reproducibility of preconception thyroid gland testing, the participants underwent two blood samplings within 2–4 weeks and an ultrasound examination of the thyroid gland. The ages ranged from 18 to 40 years. We excluded participants with irregular menstrual cycles, with manifest hyper- or hypothyroidism or when taking thyroid-specific medication, and heavy smokers (>20 cigarettes daily). Control women were allowed to remain on contraceptives. Collected serum samples were stored frozen at −72°C until assay. In each of the collected serum samples, the concentrations of TSH and anti-TPO antibodies were determined.

### Follow-up study during pregnancy and after birth

For the prospective follow-up study of the thyroid function during pregnancy and after birth, women were asked to participate during their infertility workup, prior to pregnancy. The entire cohort of patients that underwent infertility diagnostic workup consisted of 5762 women presenting in the fertility care unit of the Women’s Hospital of the University of Basel between November 2009 and September 2015. The inclusion criteria were as follows: infertile women of reproductive age (≤40 years), serum TSH levels confirmed based on two blood samples collected at an interval of 2–4 weeks, anti-TPO antibody level (based on the in-house cut-off level for normality: ≤100 IU/ml) and they were asked to refrain from nicotine abuse (≤20 cigarettes daily). Patients were assigned with subclinical hypothyroidism only in the presence of two concordant TSH results. Written informed consent to participate in the study was obtained when the results of the thyroid workup were available. Based on the serum TSH levels and on the presence or absence of circulating anti-TPO antibodies, six groups were formed:

Group 1: TSH <2.5 mIU/l and anti-TPO <100 IU/ml.

Group 2: TSH >2.5–4.5 mIU/l and anti-TPO <100 IU/ml.

Group 3: TSH > 4.5 mIU/l and anti-TPO <100 IU/ml.

Group 4: TSH <2.5 mIU/l and anti-TPO >100 IU/ml.

Group 5: TSH >2.5–4.5 mIU/l and anti-TPO >100 IU/ml.

Group 6: TSH > 4.5 mIU/l and anti-TPO >100 IU/ml.

The preconception cut-off levels of TSH are taken from the literature (Surks *et al.*, 2004; Wartofsky and Dickey, 2005). Prior to pregnancy, the patients of Groups 3 and 6 (TSH >4.5 mIU/l) were treated with thyroxine, using a daily starting dose of 50 or 75 µg and raising the dose incrementally by 25 µg until the TSH levels were in the low reference range considered as normal (0.3–2.5 mIU/l). This study was carried out in Switzerland, where since 1902, salt for cooking is iodized with 20 parts per million potassium-iodide, and since 2014 with 25 parts per million (Delange et al., 2002).

Pregnancy was defined by a serum βHCG-level >100 IU/l. After natural conception, βHCG was measured during secondary amenorrhea, or on the 14th day after ovulation or after egg retrieval (for IVF and ICSI) or embryo transfer (in thawing cycles). Timing of pregnancy was calculated from the date of the last menstruation, or from the date of ovulation + 2 weeks (if known), or from the start of ovarian stimulation with gonadotrophins or clomiphene citrate + 2 weeks, or from the date of embryo transfer in IVF or ICSI + 16 or 19 days. From the sixth to the 12th week of pregnancy, serum samples were collected weekly, and beyond the 12th week of pregnancy, monthly. At each instance, the intake of medication was noted including thyroxine. After birth, an additional serum sample was collected. The collected serum samples were stored frozen at −72°C until assay.

### Number of participants

Based on a TSH test carried out for thyroid workup, 13.9% of infertile women were previously shown to be diagnosed with subclinical hypothyroidism and 3.9% in the control group (Abalovich *et al.*, 2007). With an alpha-error rate of 5% and a beta-error rate of 5%, 175 infertile patients and controls were considered needed to achieve a statistical power of 95%.

Owing to a lack of published data, a statistical power calculation for the numbers of participants needed in the prospective follow-up study during pregnancy was not possible. Based on the data of one study in subjects in whom the long-term variations of the TSH setpoint was studied (Andersen *et al.*, 2002), 16 participants were considered to be needed in each of the six groups, giving a total of 96 pregnant individuals.

### Hormone analyses

For the prevalence study, the concentrations of both TSH and anti-TPO were measured in the serum samples at two time points. For the follow-up study during pregnancy, the concentrations of the following hormones were measured: TSH, thyroxine (T4), triiodothyronine (T3), free T4 (fT4), free T3 (fT3), anti-TPO, prolactin, βHCG, 17β-estradiol (estradiol), and progesterone. For the purpose of this study, all measurements were carried out in batches using immunoassays (ElecSys, (Roche Diagnostics, Rotkreuz, Switzerland)). The analytical accuracy of the TSH and anti-TPO assays was tested by repeat measurements of 29 serum samples. For TSH, the coefficient of variation (CV) was <5%, for anti-TPO <10%.

### Statistical analysis

Differences in the hormone concentrations were evaluated both with non-parametric tests, such as Mann–Whitney *U* and Kruskal Wallis, and with parametric tests, such as ANOVA. Differences in odds and risk ratios were compared with χ^2^ analysis. The level of statistical significance was set at the 5% level. The accuracy of repeated hormone measurements of a subset of single serum samples was compared both with linear regression analysis and with the CV, set to be below 20%. The relations between influential factors and target hormones during early and late pregnancy were evaluated by multiple linear regression analysis and the correlations were analyzed both with Student’s *t*-test and with ANOVA. The best regression models as given by covariates with statistical significance <5% are provided with their respective slope and their 95% CI. For the regression models with more than one statistically significant factor, collinearity was evaluated with the variance inflation factor (VIF). The hormone concentrations are presented as their mean values ±95% CI. The results of the statistical analyses are presented in APA-style (American Psychological Association, APA, 7th edition (2017), University of Lincoln, UK). The number of outliers of TSH, fT4, and fT3 in patient groups are binomially distributed and therefore presented by their maximum-likelihood estimation (MLE) ±95% CI. Most of the statistical analyses were carried out with the SPSS software (package 28, IBM, Armonk, NY, USA). MLE ±95% CI was calculated using an online calculator (www.ssu.shef.ac.uk).

## Results

### Prevalence study: reproducibility of repeat measurements of TSH and anti-TPO during preconception at different times

**Figure 1.**
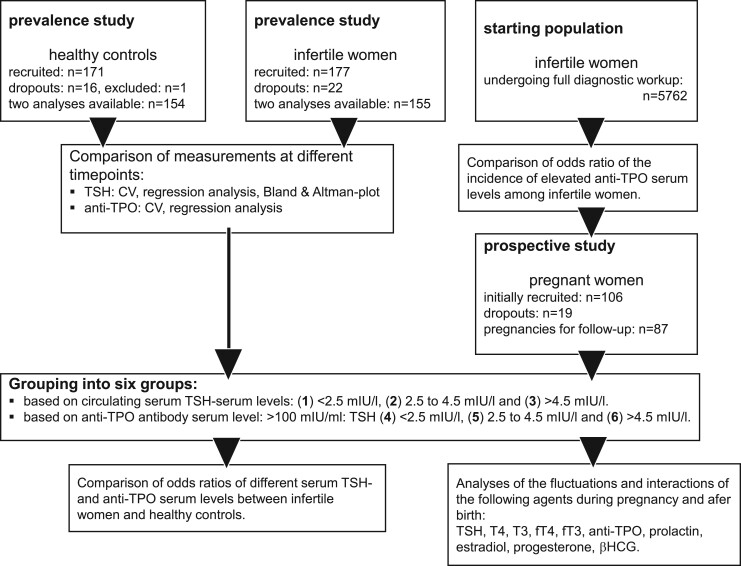
**Flow chart of two studies of thyroid hormones before and during pregnancy with four populations of infertile women and controls not aiming for pregnancy.** The prevalence of thyroid gland abnormalities was quantified in 177 infertile women as compared to 171 controls without infertility. Among a large cohort of 5762 infertile women, a total of 87 women were recruited to follow-up the status of the thyroid gland during pregnancy. TSH, thyroid-stimulating hormone; anti-TPO, thyroid peroxidase antibodies; T4, thyroxine; fT4, free thyroxine; T3, triiodothyronine; fT3, free triiodothyronine.

**Figure 2.**
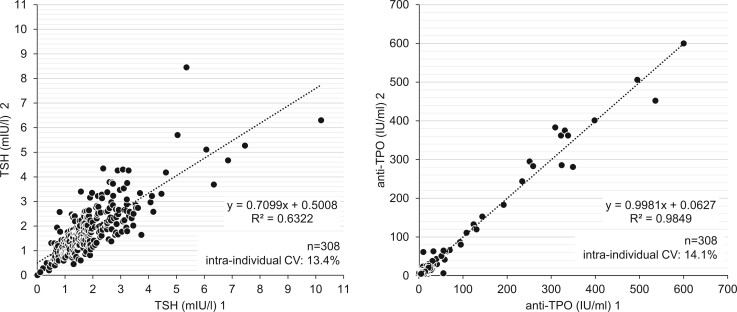
**The coherence of measurements of TSH and anti-TPO at two different time points.** Coherence was tested by two sequential measurements of the serum levels of TSH and anti-TPO, carried out within 2–4 weeks in 154 controls and in 155 infertile women and the diagnostic accuracy of both measurements was compared using linear regression analysis. TSH, thyroid-stimulating hormone; anti-TPO, thyroid peroxidase antibodies.

**Figure 3.**
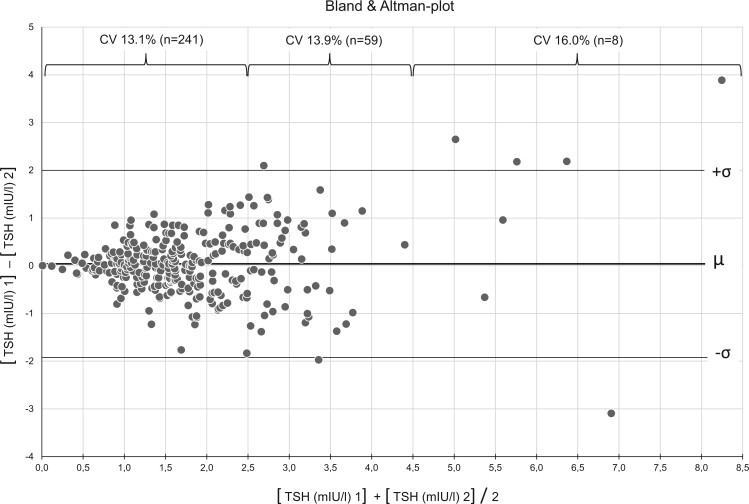
**Coefficients of variation of TSH at different concentrations were calculated to demonstrate the diagnostic accuracy of repeated TSH measurements at two different time points.** The results are presented using the Bland–Altman plot. TSH, thyroid-stimulating hormone; µ, mean value; σ, standard deviation.

Among the 177 infertile women and the 171 controls originally recruited, the serum levels of TSH and of anti-TPO of 154 infertile women and 155 control women were available at two different times, each 2–4 weeks apart (Fig. 1). Forty-eight women in the control group were taking some form of hormonal contraception. The degree of concordance was evaluated both with linear regression analyses and with the respective intra-individual CV (Fig. 2). Whereas the measurements of the anti-TPO serum levels were highly correlated (correlation coefficient *R*^2^: 0.9849), the *R*^2^ of the repeat measurements of the TSH serum levels was lower: *R*^2^ = 0.6322. The Bland and Altman plot was then used to depict the degree of concordance of TSH measurements at the given cut-off levels of TSH: at TSH levels 0.3–2.5 mIU/l, the CV was 13.1%; at TSH levels >2.5–4.5, mIU/l the CV was 13.9%; whereas at TSH levels >4.5 mIU/l, the CV was 16.0% (Fig. 3).

### Prevalence study: odds of elevated levels of TSH with or without anti-TPO among infertile women

**Figure 4.**
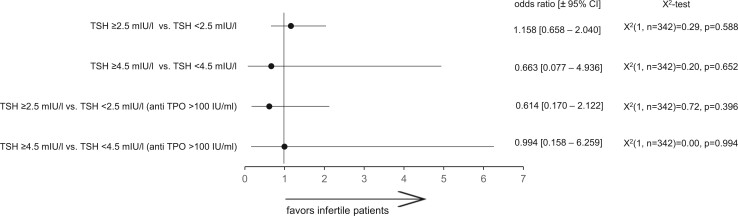
**The odds ratio of being diagnosed with subclinical hypothyroidism in infertility.** The odds ratio was calculated based on two different cut-off levels (2.5 and 4.5 mIU/l, respectively) and both with or without anti-TPO in 177 infertile women and in 171 control women. TSH, thyroid-stimulating hormone; anti-TPO, thyroid peroxidase antibodies.

The number of cases presenting with TSH levels at the given cut-off levels and with or without anti-TPO antibody levels >100 IU/ml was compared in 177 patients presenting with infertility and in 171 control women (Fig. 4). The odds of subclinical hypothyroidism, as defined by the proposed two cut-off levels of TSH, either with or without anti-TPO, were similar in either group.

### Prospective follow-up study: fluctuations in serum TSH levels during pregnancy and after birth

**Figure 5.**
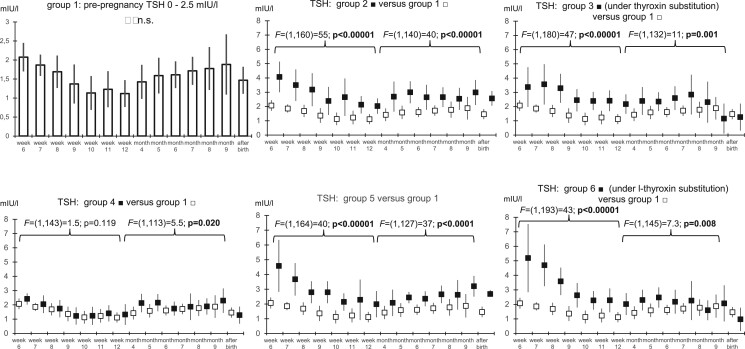
**Serum levels of TSH measured in 87 previously infertile women on a weekly basis until the 12th week of gestation, thereafter monthly, and once after delivery.** The results were grouped based on preconception TSH: Group 1 with TSH <2.5 mIU/l; Group 2 with TSH 2.5–4.5 mIU/l; Group 3 with TSH >4.5 mIU/l; Group 4 with TSH <2.5 mIU/l + anti-TPO antibody serum level >100 mIU/ml; Group 5 with TSH 2.5–4.5 mIU/l + anti-TPO antibody serum level >100 mIU/ml, and Group 6 with TSH >4.5 mIU/l + anti-TPO antibody serum level >100 mIU/ml. TSH, thyroid-stimulating hormone; anti-TPO, thyroid peroxidase antibodies.

Eighty-seven infertile women were recruited for high-frequency serum sampling throughout pregnancy and after delivery. Thirty women became pregnant in their unstimulated natural menstrual cycle (34.4%), and 57 after controlled ovarian hyperstimulation for IVF or ICSI (65.5%). Based on their preconception setpoint levels of TSH and on the presence or absence of anti-TPO antibodies, they were divided into six groups. The clinical characteristics of the participating pregnant women are presented in Table 1. The serum TSH levels of the pregnant women with preconception serum TSH levels between 0.3 and 2.5 mIU/l and with anti-TPO antibody levels ≤100 IU/ml decreased during early pregnancy, but the differences did not reach statistical significance (Fig. 5). In general, when comparing the serum levels of TSH to those of Group 1, the serum levels of TSH in Groups 2–6 were significantly higher, particularly in women carrying anti-TPO antibodies (*P* < 0.02 to *P* < 0.00001, Groups 4–6) and in women substituted with thyroxine (*P* < 0.001 to *P* < 0.00001, Groups 3 and 6). After birth, the serum levels of TSH were lower in all groups, but the differences did not reach statistical significance.

**Table 1.** Baseline characteristics of healthy controls and the infertile patients recruited to study the prevalence of subclinical hypothyroidism, and of the previously infertile patients recruited for follow-up during pregnancy.

	No.	Age (y.)	Age at menarche (y.)	Duration of menstrual cycle (d.)	BMI (kg/m^2^)	RR systolic (mm Hg)	RR diastolic (mm Hg)	Heartbeat frequency (per min)	Baseline TSH (mIU/l)	Baseline anti-TPO (IU/l)
Prevalence study										
Infertile patients	155	33.7 ± 0.7	13.3 ± 0.3	28.1 ± 0.9	23.0 ± 0.6	122.1 ± 2.1	72.0 ± 0.4	72.0 ± 1.6	1.94 ± 1.50	22.7 ± 8.5
Healthy controls	154	32.3 ± 0.9	13.0 ± 0.2	27.5 ± 0.4	23.2 ± 0.6	124.9 ± 1.9	73.4 ± 1.2	72.5 ± 1.6	1.73 ± 1.20	47.7 ± 18.0
ANOVA		***F*(1, 306) = 5.7** *P*** = 0.018**	*F*(1, 306) = 2.8 *P* = 0.094	*F*(1, 306) = 1.3 *P* = 0.252	*F*(1, 306) = 0.1 *P* = 0.722	*F*(1, 306) = 3.6 *P* = 0.058	*F*(1, 306) = 1.8 *P* = 0.182	*F*(1, 306) = 1.8 *P* = 0.182	*F*(1, 306) = 0 *P* = 0.840	***F*(1, 306)** = **3.9** *P*** = 0.049**
Pregnancy follow-up study										
Group 1	17	33.4 ± 2.1	13.4 ± 0.7	30.6 ± 1.9	24.9 ± 2.2	123.3 ± 5.2	74.2 ± 3.7	73.5 ± 4.9	1.5 ± 0.2	10.1 ± 0.2
Group 2	14	32.9 ± 1.5	12.9 ± 0.9	33.9 ± 8.6	23.8 ± 1.9	124.6 ± 7.2	75.6 ± 5.2	75.2 ± 8.2	3.4 ± 0.3	11.3 ± 2.5
Group 3	17	32.8 ± 2.0	12.9 ± 0.9	28.5 ± 1.2	23.7 ± 2.5	117.8 ± 5.3	70.8 ± 3.6	72.7 ± 4.7	5.9 ± 0.6	13.3 ± 3.6
Group 4	9	31.0 ± 2.9	13.4 ± 1.2	35.2 ± 13.4	25.2 ± 4.7	123.7 ± 10.6	72.4 ± 5.7	74.9 ± 6.9	2.1 ± 0.4	264 ± 199
Group 5	12	33.0 ± 2.8	13.1 ± 1.0	30.0 ± 2.8	23.6 ± 1.9	124.3 ± 7.3	74.3 ± 5.5	72.9 ± 3.1	3.8 ± 0.6	429 ± 172
Group 6	16	33.9 ± 2.0	13.5 ± 0.8	29.4 ± 2.4	21.8 ± 1.6	118.9 ± 7.3	74.3 ± 5.1	73.9 ± 4.6	8.0 ± 2.1	1035 ± 506
ANOVA		*F*(5, 79) = 0.6 *P* = 0.710	*F*(5, 79) = 0.4 *P* = 0.862	*F*(5, 79) = 0.9 *P* = 0.501	*F*(5, 79) = 0.2 *P* = 0.990	*F*(5, 79) = 0.8 *P* = 0.562	*F*(5, 79) = 0.5 *P* = 0.742	*F*(5, 79) = 0.1 *P* = 0.987	***F*(5, 79)** = **22.5** *P*** < 0.00001**	***F*(5, 79)** = **11.4** *P*** < 0.00001**

Group 1: TSH <2.5 mIU/l and anti-TPO <100 IU/ml.

Group 2: TSH >2.5–4.5 mIU/l and anti-TPO <100 IU/ml.

Group 3: TSH > 4.5 mIU/l and anti-TPO <100 IU/ml.

Group 4: TSH <2.5 mIU/l and anti-TPO >100 IU/ml.

Group 5: TSH >2.5–4.5 mIU/l and anti-TPO >100 IU/ml.

Group 6: TSH > 4.5 mIU/l and anti-TPO >100 IU/ml.

TSH, thyroid-stimulating hormone; anti-TPO, thyroid peroxidase antibodies; RR: risk ratio.

If the cut-off value of TSH was set at 4.5 mIU/l, the number of out-of-range TSH values during ongoing pregnancies was higher in patients included in Groups 2, 3, 5, and 6 (Table 2). If, however, the cut-off value of TSH was set at 2.5 mIU/l, the number of out-of-range TSH values during ongoing pregnancy was much higher and significantly elevated in all five patient groups, with four times higher χ^2^-values than if the TSH cut-off value was set at 4.5 mIU/l (Table 2).

**Table 2.** Outliers of TSH during pregnancies in the six groups defined by preconception TSH.

Group	Total no.	TSH >2.5 mIU/l	MLE	95% CI	χ^2^	*P* value^1^	TSH >4.5 mIU/l	MLE^2^	95% CI^1^	χ^2^	*P*-value^1^
1	178	15					1				
2	148	72	5.7	2.5–10.0	66.8	<0.0001	18	5.3	0.3–24.0	17.5	<0.0001
3	157	68	5.1	3.2–8.9	54.4	<0.0001	19	5.4	0.3–24.1	17.5	<0.0001
4	102	16	1.9	1.0–3.6	3.5	=0.002	0				
5	137	61	5.2	3.2–9.2	55.1	<0.0001	15	5.9	0.3–27.2	15.5	<0.001
6	187	92	5.8	3.6–10.0	73.2	<0.0001	29	4.2	0.2–18.2	21.3	<0.0001

TSH, cut-off values >2.5 or >4.5 mIU/l.

1 Based on Chi-squared analysis (χ^2^).

2 Equal-tailed maximum-likelihood estimation (MLE) and 95% CI comparison of binomial risk ratios.

Group 1: TSH <2.5 mIU/l and anti-TPO <100 IU/ml.

Group 2: TSH >2.5–4.5 mIU/l and anti-TPO <100 IU/ml.

Group 3: TSH > 4.5 mIU/l and anti-TPO <100 IU/ml.

Group 4: TSH <2.5 mIU/l and anti-TPO >100 IU/ml.

Group 5: TSH >2.5–4.5 mIU/l and anti-TPO >100 IU/ml.

Group 6: TSH > 4.5 mIU/l and anti-TPO >100 IU/ml.

TSH, thyroid-stimulating hormone.

### Prospective follow-up study: fluctuations in serum levels of T4, T3, fT4, fT3, and prolactin during pregnancy and after birth

**Figure 6.**
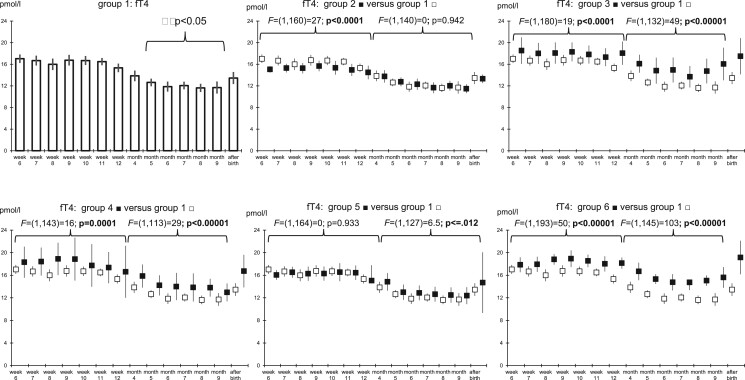
**Serum levels of fT4 measured in 87 previously infertile women on a weekly basis until the 12th week of gestation, thereafter monthly, and once after delivery.** The results were grouped based on preconception TSH. Group 1 with TSH <2.5 mIU/l; Group 2 with TSH 2.5–4.5 mIU/l; Group 3 with TSH >4.5 mIU/l; Group 4 with TSH <2.5 mIU/l + anti-TPO antibody serum level >100 mIU/ml; Group 5 with TSH 2.5–4.5 mIU/l + anti-TPO antibody serum level >100 mIU/ml, and Group 6 with TSH >4.5 mIU/l + anti-TPO antibody serum level >100 mIU/ml. TSH, thyroid-stimulating hormone; anti-TPO, thyroid peroxidase antibodies; fT4, free thyroxine.

The fluctuations of the serum levels of the major thyroid hormones, T4, T3, fT4, and fT3 were measured during ongoing pregnancies and after birth in the six predefined patient groups (Fig. 6; Supplementary Figs S1, S2, and S3). Similarly, the changes in the serum levels of prolactin were measured and taken as reference (Supplementary Fig. S4). Whereas in Group 1, the serum levels of T4 and T3 remained virtually constant during pregnancy (Supplementary Figs S1 and S2), the serum levels of fT4 and fT3 initially remained constant, but then underwent a decline starting in the 5th month of gestation until delivery (Fig. 6; Supplementary Fig. S3, *P* < 0.05). In Groups 2–5, the serum level patterns of all four thyroid hormones closely followed the patterns of Group 1. The patients included in Groups 3 and 6 were substituted with thyroxine resulting in significantly higher levels of T4 and fT4 (Supplementary Fig. S1; Fig. 6). In general, the serum levels of T4 and fT4 were similar or higher than those observed in Group 1, whereas those of T3 and fT3 were similar or lower (Group 4) than those observed in Group 1. After birth, the serum levels of T4 and T3 declined in all groups, whereas those of fT4 and fT3 remained similar to those during pregnancy.

Using established normal laboratory range values of fT4 (for fT4, see also Laurberg *et al.*, 2016) and fT3, we determined out-of-range values in the five patient groups and compared the number of out-of-range values in each group with that of reference group 1 (Supplementary Tables S1 and S2). The total number of samples with low fT4 (<11.6 pmol/l) was 46 (6.3%), whereas the total number of samples with high fT4 (>22.0 pmol/l) was 24 (3.3%) (Supplementary Table S1). Significantly fewer samples with low fT4 were detected in the patients included in Groups 3 and 6 (both substituted with thyroxine) and in Group 4 (normal preconception TSH but with anti-TPO antibodies). Furthermore, there were no samples with low fT3 (<2.6 pmol/l), while the total number of samples with high fT3 (>22.0 pmol/l) was 44 (6.0%). The number of serum samples with high fT3 in each of the five groups was similar to the number of serum samples with high fT3 in reference group 1 (Supplementary Table S2).

In pregnant patients of all groups, the serum levels of prolactin underwent a significant rise and values dropped after delivery (Supplementary Fig. S4). Although some minor differences in the prolactin levels of Groups 2, 4, and 6 were present, the changes in serum prolactin levels generally followed the pattern of the pregnant women included in reference group 1.

### Prospective follow-up study: changes in the serum levels of anti-TPO during pregnancy

**Figure 7.**
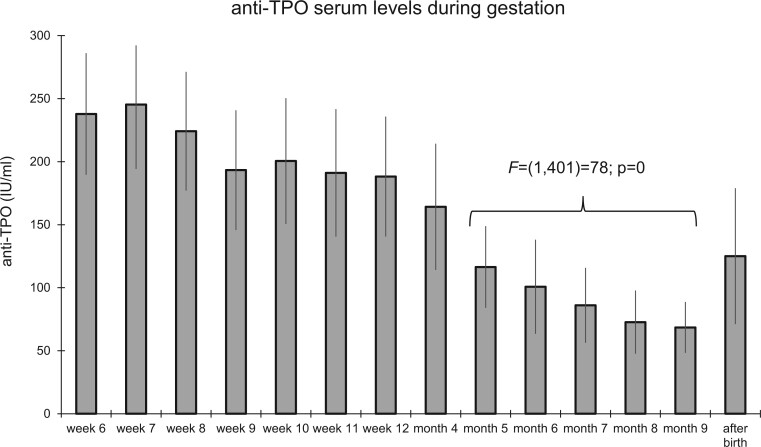
**Serum levels of anti-TPO in 74 previously infertile patients with preconception anti-TPO >100 mIU/ml determined throughout pregnancy (Groups 4–6).** A significant drop in the serum levels of anti-TPO was observed from the fifth month of gestation onward (*P* < 0.0001). After delivery, the serum level of anti-TPO increased again. anti-TPO, thyroid peroxidase antibodies.

Seventy-four previously infertile women were diagnosed with anti-TPO antibody levels >100 IU/l and were followed up during pregnancy (Table 1). The serum levels of anti-TPO antibodies underwent a pronounced decline during pregnancy (Fig. 7), which reached the level of statistical significance from the fifth month of gestation onward (*P* < 0.0001). After birth, the serum levels of anti-TPO rose again to preconception levels.

### Prospective follow-up study: influential factors involved in modifying the serum levels of TSH, fT4, fT3, and prolactin during early and late pregnancy

We labeled βHCG, estradiol, progesterone, and anti-TPO as influential variables, that may potentially influence the endocrine function of the thyroid gland. TSH, fT4, fT3, and prolactin were labeled target parameters, which may be affected by one or more of the influential variables during pregnancy. The relation between the influential variables and the target parameters during both early (gestational weeks 6–9) and late pregnancy (gestational months 6–9) were analyzed using multiple linear regression (Table 3). Durbin–Watson tests were carried out to exclude auto-correlation and collinearity between influential factors and quantified with VIF. During early pregnancy, the circulating levels of TSH were positively correlated with anti-TPO (*P* < 0.001) and with estradiol (*P* = 0.025), and negatively with βHCG (*P* < 0.001). During early pregnancy, the serum levels of fT4 were negatively correlated with the progesterone serum concentration (*P* = 0.024), whereas those of fT3 were negatively correlated with the anti-TPO serum levels (*P* = 0.007).

**Table 3.** Factors influencing thyroid gland function during pregnancy.

Response variable	Gestational period	Predictor variable	*B* (slope)	Std. error	*t*	Stand. coefficient (β)	95% CI for *B*	*P*-value	VIF (1)	DW (2)	*R* ^2^ (%) (3)	ANOVA (4)
TSH	wk. 6–9	Intercept	3.792	0.501	7.566		2.806	=4.779		1.575	10.6	*F*(3, 278) = 11.0 *P* < 0.001
		Anti-TPO	0.003	0.001	4.005	0.228	0.002–0.005	<0.001	1.08			
		βHCG	0.000	0.000	−3.735	−0.212	0.000–0.000	<0.001	1.24			
		Estradiol	7.462E−5	0.000	2.259	0.129	0.000–0.000	=0.025	2.59			
	mo. 6–9	Intercept	0.987	0.535	1.843		−0.068 to 2.042	=0.067		1.612	2.4	*F*(1, 225) = 5.4 *P* = 0.021
		Progesterone	0.007	0.003	2.331	0.154	0.001–0.013	=0.021				
fT4	wk. 6–9	Intercept	18.024	0.358	50.277		17.318–18.730	<0.001		1.599	1.8	*F*(1, 280) = 5.1 *P* = 0.024
		Progesterone	−0.006	0.003	−2.268	−0.134	−0.011 to −0.001	=0.024				
	mo. 6–9	Intercept	16.876	0.976	17.473		14.953–18.800	<0.001		1.686	13.6	*F*(2, 225) = 17.7 *P* < 0.001
		Progesterone	−0.023	0.005	−4.322	−0.269	−0.033 to 0.012	<0.001	1.00			
		Anti-TPO	0.010	0.002	3.953	0.246	0.005–0.015	<0.001	1.00			
fT3	wk. 6–9	Intercept	4.895	0.045	109.179		4.807–4.983	<0.001		1.745	2.6	*F*(1, 280) = 7.5 *P* = 0.007
		Anti-TPO	−0.001	0.000	−2.738	−0.162	−0.001 to 0.000	=0.007				
	mo. 6–9	Intercept	4.443	0.209	21.231		4.031–4.856	<0.001		2.000	2.3	*F*(1, 225) = 5.3 *P* = 0.023
		Progesterone	−0.003	0.001	−2.291	−0.151	−0.005 to 0.000	=0.023				
Prolactin	wk. 6–9	Intercept	586.698	144.331	4.065		302.578–870.819	<0.001		1.808	26.0	*F*(3, 278) = 32.6 *P* < 0.001
		Estradiol	0.089	0.026	3.440	0.287	0.038–0.140	<0.001	2.88			
		Progesterone	4.092	1.518	2.697	0.226	1.105–7.080	=0.007	2.88			
		Anti-TPO	−0.885	0.386	−2.291	−0.119	−1.646 to −0.125	=0.023	1.00			
	mo. 6–9	Intercept	2777.394	899.877	3.086		1004.086–4550.702	=0.002		2.076	10.7	*F*(2, 224) = 13.4 *P* < 0.001
		Progesterone	23.506	5.082	4.625	0.305	13.491–33.520	<0.001	1.88			
		Estradiol	−0.123	0.035	−3.532	−0.233	−0.192 to −0.054	<0.001	1.88			

(1) VIF denominates the variance inflation factor (VIF = 1/(1 − *R*^2^)), which is a marker of collinearity. A value of 1 indicates no collinearity. Values up to 5 indicate moderate collinearity.

(2) DW denominates the result of the Durbin–Watson test, which should optimally be between 1.5 and 2.5.

(3) *R*^2^ denominates the coefficient of multiple determination.

(4) ANOVA findings are reported in APA format: *F* (regression degrees of freedom, residual degree of freedom) = *F*-ratio.

TSH, thyroid-stimulating hormone; fT4, free thyroxine; fT3, free triiodothyronine.

During late pregnancy, the circulating levels of TSH were positively correlated with the serum levels of progesterone (*P* = 0.021), whereas those of fT4 and fT3 were negatively correlated with the serum levels of progesterone (*P* < 0.001 and *P* = 0.023, respectively). In contrast, during late pregnancy, the serum levels of fT4 were positively correlated with the serum levels of anti-TPO (*P* < 0.001).

Overall, the regression models that best represented the observed data were that of prolactin (*R*^2^ = 26.0% during early pregnancy and *R*^2^ = 10.7% during late pregnancy), that of fT4 during late pregnancy (*R*^2^ = 13.6%) and that of TSH during early pregnancy (*R*^2^ = 10.6%). All other regression models had low levels of prediction (<5%).

## Discussion

Whereas there is no doubt that overt hypothyroidism must be substituted with thyroxine, particularly in women trying to conceive, the TSH cut-off limits of what defines subclinical hypothyroidism have been a matter of debate (Surks, 2013; Brabant *et al.*, 2015). Anti-TPO antibodies are involved in worsening the condition of thyroid function (Glinoer *et al.*, 1994) and are associated with complications during pregnancy (Poppe and Glinoer, 2003). Thyroxine substitution has been advocated at lower threshold levels of TSH in fertility care, in the presence of anti-TPO antibodies and in repeated miscarriage (Alexander *et al.*, 2017; Poppe *et al.*, 2021). However, recent randomized clinical trials have not demonstrated higher live birth numbers resulting from treatment with thyroxine in euthyroid women carrying anti-TPO (Dhillon-Smith *et al.*, 2019), even in cases with recurrent pregnancy loss (Van Dijk *et al.*, 2022).

We aimed to bridge the data gap between preconception TSH serum level setpoints (with and without anti-TPO) and the dynamic changes in thyroid endocrine function during the subsequent pregnancy. First, we studied in infertile women the accuracy and the reproducibility of preconception thyroid gland screening based on TSH and anti-TPO. Dual measurements of TSH and anti-TPO at different time points during preconception provided concordant results, as shown by linear regression analysis (Fig. 2), despite a higher CV (16.0%) at TSH serum levels >4.5 mIU/l (Fig. 3). The odds of being diagnosed with subclinical hypothyroidism at both TSH threshold levels (either 2.5 or 4.5 mIU/l, with or without anti-TPO) were similar among infertile women and control women not aiming for pregnancy (Fig. 4). In an observational cohort study among infertile women and women with a history of miscarriage in the UK, the prevalence of overt hypothyroidism was 0.2%; of subclinical hypothyroidism with serum TSH levels above 4.5 mIU/l, 2.4%; and of subclinical hypothyroidism with serum TSH levels between 2.5 and 4.5 mIU/l, 17.6% (Dhillon-Smith *et al.*, 2020).

Whereas the serum levels of TSH generally decreased during early pregnancy, the levels in the various subgroups during pregnancy corresponded to their preconception values, even in those substituted with thyroxine (Groups 3 and 6). Notwithstanding higher serum levels of TSH in cases with higher preconception setpoints of TSH (Fig. 5), the serum levels of T4, T3, fT4, and fT3 remained within or slightly higher than those in the reference group 1 (Fig. 6; Supplementary Figs S1, S2, and S3) rendering undertreatment unlikely. During pregnancy, lower threshold levels of TSH are considered to be needed to initiate supplementation with thyroxine (Laurberg *et al.*, 2016), because HCG competitively binds TSH receptors generally resulting in lower TSH (Pekonen *et al.*, 1988). To quantify the involvement of influential factors regulating thyroid function during early pregnancy, we examined the interdependencies of hCG, estradiol, progesterone, and anti-TPO with TSH and thyroid gland function. For comparison, we used the well-established linear relation between prolactin and estradiol during pregnancy (Rigg *et al.*, 1977) as a control (Table 3). As expected, multiple linear regression analyses confirmed that estradiol concentrations positively correlated with the prolactin levels circulating during early pregnancy (β = 0.287). Interestingly, during late pregnancy, instead of estradiol (β = −0.233), progesterone positively correlated with the circulating prolactin levels (β = 0.305), but there was some collinearity between the progesterone and estradiol serum levels during pregnancy (VIF between 1.88 and 2.88, Table 3). In general, the regression models predicted the serum levels of prolactin better than hormones of the thyroid gland (Table 3). Therefore, the modulating effects of high circulating estradiol and HCG levels and of anti-TPO on the endocrine output of the thyroid gland during pregnancy appear to be of lesser importance than the relation between the serum levels of prolactin and estradiol, which was taken as a reference here. From these comparisons, the robust individual setpoint of TSH described earlier (Andersen *et al.*, 2002) seems decisive in determining the circulating TSH levels, even during pregnancy.

Measuring the endocrine output of thyroid hormones themselves provides an alternative to TSH. During pregnancy, the fractions of free circulating thyroid hormones are reduced by the higher estrogen-mediated hepatic production and secretion of binding proteins into the blood circulation (Doe *et al.*, 1967). Despite this, and despite generally higher serum levels of TSH during early pregnancy in cases diagnosed with high preconception setpoints of TSH (Fig. 5), even in those substituted with thyroxine (Groups 3 and 6), in all subgroups the serum levels of T4, T3, fT4, and fT3 were similar to, or even somewhat higher (not lower) than, those in the reference group 1 (Fig. 6, Supplementary Figs S1, S2, and S3). Only a few outlying fT4 and fT3 values were observed (Supplementary Table S1). The lower serum levels of the free fractions from the fifth month of gestation onward (*P* < 0.05, Fig. 6, Supplementary Fig. S3) correspond to earlier findings (McClain *et al.*, 2008).

The serum levels of anti-TPO also underwent a significant decline during pregnancy, an observation recently described elsewhere (Li *et al.*, 2020). The mechanisms involved are yet to be identified, but the decline occurred at a time during pregnancy when the placenta is known to actively manage the passage of immunoglobulins between the mother and the fetus (Malek *et al.*, 1998; Simister, 2003). At birth, the serum levels of anti-TPO in both maternal and cord blood are highly correlated (Seror *et al.*, 2014). The progressive decline of anti-TPO serum levels during ongoing pregnancies may also constitute a protective element toward the maternal thyroid gland. Recently, and in agreement, ESHRE guidelines revised their earlier recommendation to provide thyroxine to euthyroid pregnant women carrying anti-TPO (ESHRE Guideline Group on RPL *et al.*, 2023).

The main strengths of the present study include the following: the assessment of the reproducibility of conventional diagnostic testing of thyroid function before conception among infertile women; the assessment of different threshold levels of preconception TSH setpoints with and without anti-TPO antibodies on the endocrine performance of the thyroid gland during subsequent pregnancy and after birth; and the many timepoints of endocrine assessment during pregnancy using multiple parameters of thyroid function together with candidate influential factors of thyroid function, such as HCG and anti-TPO, thereby using prolactin as reference hormone with well-established fluctuations of its serum levels during pregnancy.

This analysis of thyroid function from preconception to pregnancy and after birth has limitations. Although the number of participants, both in the preconception and in the pregnancy follow-up study, was estimated based on a preliminary calculation of statistical power, the number of participants in our academic single center institution remained limited owing to compliance restraints of the participating individuals. The actual number of participants with dual measurements in the prevalence part of the study was 310 instead of the 350 estimated to be needed. For similar reasons, the number of pregnant participants in the follow-up study was 87 instead of the 96 estimated to be needed. The time needed to recruit the pregnant women included in the pregnancy follow-up study lasted nearly 6 years, whereas the number of dropouts during the various phases of pregnancy and after delivery was considerable (909 measurements instead of the foreseen 1218, 74.6%). Furthermore, a proportion of the pregnancies, that were included in the follow-up study, arose after assisted reproduction, which entail higher levels of sex steroids during early pregnancy (De Geyter *et al.*, 2009) and which influence thyroid function differently than in naturally conceived pregnancies (Poppe *et al.*, 2004; Velkeniers *et al.*, 2013). However, this latter limitation is reduced by the low prediction of the free thyroid hormones by the circulating estradiol levels, as given by the multiple regression analysis (Table 3). Finally, for ethical reasons, infertile women with preconception TSH >4.5 mIU/l were substituted with thyroxine.

We conclude that the odds of being diagnosed with subclinical hypothyroidism, with or without anti-TPO antibodies, were similar in infertile women versus comparable aged controls currently not aiming for pregnancy, regardless of lower or higher threshold values of TSH. In infertile women with preconception TSH 2.5–4.5 mIU/l, the circulating TSH levels during early pregnancy reflected those measured during preconception. Even infertile women with preconception TSH >4.5 mIU/l and substituted with thyroxine presented with circulating TSH levels during early pregnancy similar to those in preconception, despite a normal or slightly elevated thyroid hormone output. These findings suggest that TSH may not be the best parameter of thyroid function during pregnancy. During pregnancy, deviations of TSH from the proposed preconception cut-off values have minimal or no impact on the circulating levels of T4, T3, fT4, or fT3. Free T4-serum levels may better reflect thyroid function in pregnant women previously diagnosed with subclinical hypothyroidism. In addition, during pregnancy, serum fT4 levels reflect adherence to exogenous treatment with thyroxine better than TSH. The newly proposed reference levels of fT4 during pregnancy will be beneficial to promote the use of fT4 as a marker of thyroid function during pregnancy (Osinga *et al.*, 2022). The adoption of these measures will hopefully contribute to limit the current overuse of thyroxine in fertility care.

## Supplementary Material

hoad038_Supplementary_Figure_S1

hoad038_Supplementary_Figure_S2

hoad038_Supplementary_Figure_S3

hoad038_Supplementary_Figure_S4

hoad038_Supplementary_Table_S1

hoad038_Supplementary_Table_S2

## Data Availability

The data that support the findings are available on request from the corresponding author, upon reasonable request.
