# Supplementary material for: In infertile women with subclinical hypothyroidism, with or without thyroid peroxidase antibodies, serum TSH during pregnancy follows preconception values and thyroid hormones remain stable
Source: Hum Reprod Open. 2023 Oct 9;2023(4):hoad038. doi: 10.1093/hropen/hoad038 (PMC10589916; doi:10.1093/hropen/hoad038)
Supplement: hoad038_Supplementary_Figure_S3 [file hoad038_supplementary_figure_s3.pdf]

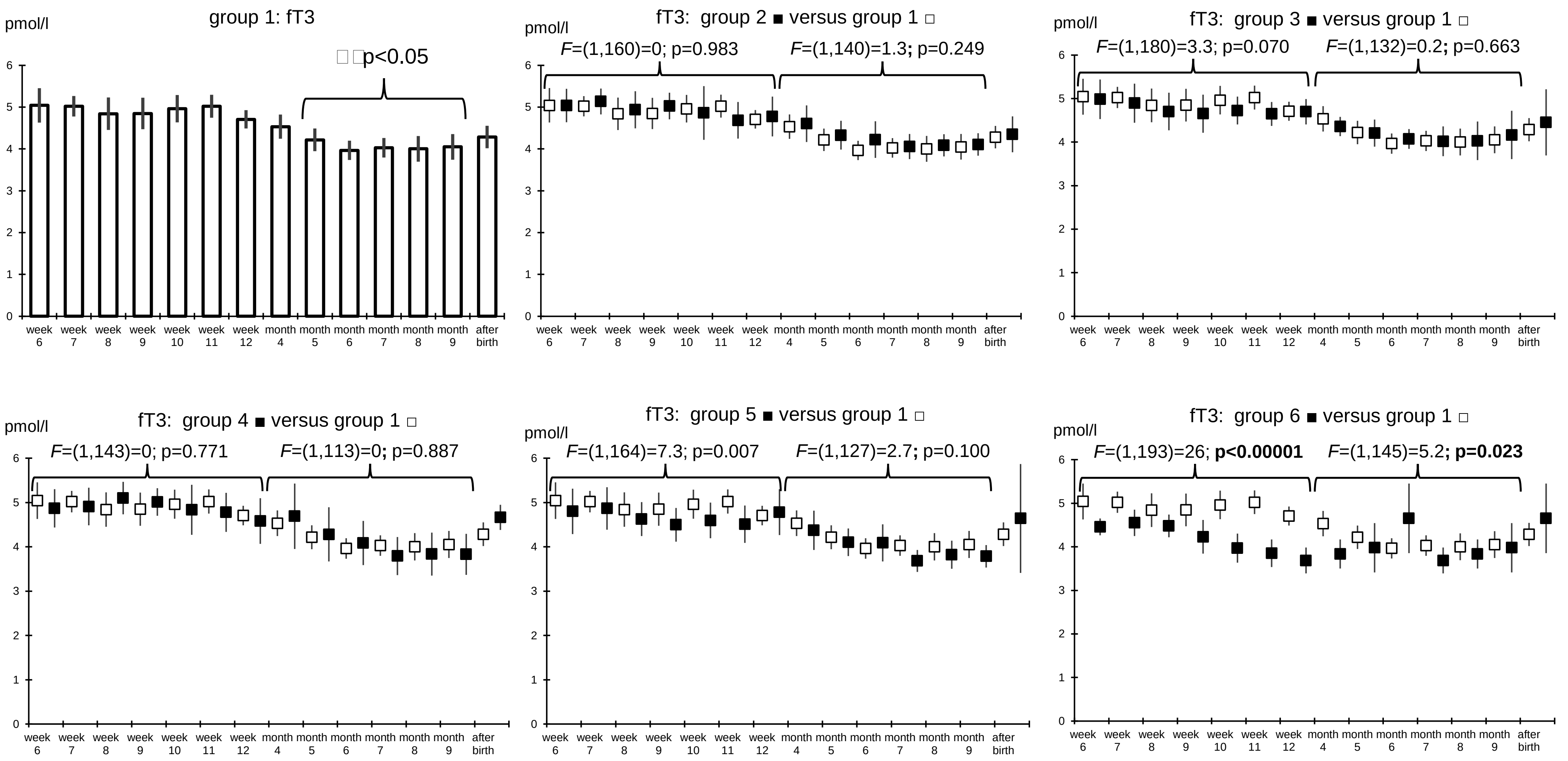

Supplementary Figure S3: Serum free T3 (fT3) throughout pregnancy and after delivery.

Serum levels of fT3 were measured in 87 previously infertile women on a weekly basis until the 12th week of gestation, thereafter monthly, and once after delivery. The results were grouped into six based on preconception TSH (see Figure 5).
