# Supplementary material for: In infertile women with subclinical hypothyroidism, with or without thyroid peroxidase antibodies, serum TSH during pregnancy follows preconception values and thyroid hormones remain stable
Source: Hum Reprod Open. 2023 Oct 9;2023(4):hoad038. doi: 10.1093/hropen/hoad038 (PMC10589916; doi:10.1093/hropen/hoad038)
Supplement: hoad038_Supplementary_Table_S1 [file hoad038_supplementary_table_s1.docx]

**Supplementary Table S1:** Free thyroxine outside the normal limits during pregnancy.

| group | total no. | fT4  <11.6 pmol/l | MLE | 95% CI | X^2^ | p-  value^2^ | fT4  >22.0 pmol/l | MLE^1^ | 95% CI^1^ | X^2^ | p-  value^2^ |
| --- | --- | --- | --- | --- | --- | --- | --- | --- | --- | --- | --- |
| 1 | 178 | 20 |  |  |  |  | 0 |  |  |  |  |
| 2 | 148 | 23 | 1.4 | 0.8–2.4 | 1.3 | =0.253 | 0 |  |  |  |  |
| 3 | 157 | 4 | 0.2 | 0.1–0.6 | 8.6 | **=0.003** | 9 |  |  |  |  |
| 4 | 102 | 1 | 0.1 | 0.005–0.4 | 8.7 | **=0.003** | 10 |  |  |  |  |
| 5 | 137 | 17 | 1.1 | 0.6–2.0 | 0.1 | =0.749 | 1 |  |  |  |  |
| 6 | 187 | 1 | 0.1 | 3.003–0.02 | 18.8 | **<0.0001** | 4 |  |  |  |  |

fT4: free thyroxine

1. Equal-tailed maximum-likelihood estimation (MLE) and 95% confidence intervals for comparison of binomial risk ratios.
2. Based on Chi-squared analysis (X^2^).
