# Supplementary material for: In infertile women with subclinical hypothyroidism, with or without thyroid peroxidase antibodies, serum TSH during pregnancy follows preconception values and thyroid hormones remain stable
Source: Hum Reprod Open. 2023 Oct 9;2023(4):hoad038. doi: 10.1093/hropen/hoad038 (PMC10589916; doi:10.1093/hropen/hoad038)
Supplement: hoad038_Supplementary_Table_S2 [file hoad038_supplementary_table_s2.docx]

**Supplementary Table S2:** Free triiodothyronine outside the normal limits during pregnancy.

| group | total no. | fT3  <2.6 pmol/l | MLE | 95% CI | X^2^ | p-  value^2^ | fT3  >22.0 pmol/l | MLE^1^ | 95% CI^1^ | X^2^ | p-  value^2^ |
| --- | --- | --- | --- | --- | --- | --- | --- | --- | --- | --- | --- |
| 1 | 178 | 0 |  |  |  |  | 5 |  |  |  |  |
| 2 | 148 | 0 |  |  |  |  | 12 | 2.8 | 1.1–8.7 | 4.5 | =0.321 |
| 3 | 157 | 0 |  |  |  |  | 8 | 1.8 | 0.6–5.8 | 1.2 | =0.280 |
| 4 | 102 | 0 |  |  |  |  | 8 | 2.8 | 0.9–8.9 | 3.6 | =0.591 |
| 5 | 137 | 0 |  |  |  |  | 11 | 2.8 | 1.0–8.7 | 4.3 | =0.279 |
| 6 | 187 | 0 |  |  |  |  | 5 | 1.0 | 0.2–3.4 | 0.008 | =0.930 |

fT3: free triiodothyronine

1. Equal-tailed maximum-likelihood estimation (MLE) and 95% confidence intervals for comparison of binomial risk ratios.
2. Based on Chi-squared analysis (X^2^).
